# A New Insight into Imaging Diagnosis of Otosclerosis Enhanced by Machine Learning and Radiomics

**DOI:** 10.1007/s10278-026-01843-0

**Published:** 2026-01-30

**Authors:** Marta Álvarez de Linera-Alperi, Juan Miranda Bautista, David Corral Fontecha, Josué Fernández Carnero, José A Vega, Pablo Menéndez Fernández-Miranda

**Affiliations:** 1 Otorrinolaringology Department, Hospital Universitario Rey Juan Carlos, Madrid, Spain; 2 Radiology Department, Hospital Universitario Rey Juan Carlos, Madrid, Spain; 3 Radiology Department, Complejo Asistencial Universitario de León, León, Spain; 4 Cognitive Neuroscience, Pain, and Rehabilitation Research Group (NECODOR), Rey Juan Carlos University, Madrid, Spain; 5 Department of Physical Therapy, Occupational Therapy, Rehabilitation and Physical Medicine, Rey Juan Carlos University, Madrid, Spain; 6 La Paz Hospital Institute for Health Research, IdiPAZ, Madrid, Spain; 7 Musculoskeletal Pain and Motor Control Research Group, Faculty of Sport Sciences, Universidad Europea de Madrid, Madrid, Spain; 8 Departamento de Morfología y Biología Celular, Universidad de Oviedo, Oviedo, Spain; 9 Facultad de Ciencias de La Salud, Universidad Autónoma de Chile, Santiago, Chile; 10 Escuela Politécnica Superior,Universidad CEU San Pablo, Madrid, Spain; 11 Instituto de Investigación Sanitaria Fundación Jimenez Díaz, Madrid, Spain

**Keywords:** Otosclerosis, Machine learning, Radiomics, Imaging biomarkers, Temporal bone computed tomography

## Abstract

Otosclerosis is a disease affecting the middle and inner ear, characterized by abnormal bone remodeling that leads to stapes fixation and progressive hearing loss. Although high-resolution computed tomography (HRCT) is the standard imaging modality for diagnosis, its sensitivity is limited, with a high false-negative rate (FNR). This study investigates the use of radiomics and machine learning (ML) to improve diagnostic accuracy. HRCT scans from 99 subjects (48 otosclerosis, 51 controls) were analyzed, focusing on the stapes, antefenestral region (AF), and oval window (OW). From each scan, 6048 radiomic features were extracted and reduced to 1317 through feature selection. Statistical analyses and ML modeling were performed using the selected features. Sixty-seven biomarkers showed significant differences between cases and controls, primarily in the AF (56) and stapes (11); none were found in the OW. Both the AF and stapes exhibited increased heterogeneity in otosclerosis, reflecting the bone remodeling process. A reduction in the stapes' major axis was also observed, possibly related to torsional deformation. Image transformation filters enhanced disease visibility. Among several ML classifiers tested, L2-regularized logistic regression performed best, achieving an AUC of 0.90 ± 0.06, thereby enhancing the diagnostic accuracy reported in some studies for radiologists. Hierarchical clustering of the most predictive features further confirmed their strong discriminative power. Our findings highlight the potential of radiomics and ML to standardize otosclerosis diagnosis, reduce FNR, and support surgical decision-making. Future studies should validate these results using larger cohorts and advanced imaging technologies such as Photon-Counting CT.

**Supplementary Information:**

The online version contains supplementary material available at https://doi.org/10.1007/s10278-026-01843-0.

## Introduction

Otosclerosis is a middle and inner ear disorder characterized by abnormal bone remodeling, resulting in progressive hearing loss [1–8]. In its early stage, known as otospongiosis, normal compact bone of the otic capsule and the stapes is replaced by irregular, vascularized spongy bone. As the disease advances, these areas lose vascularity, become denser, and progress to otosclerosis. This pathological process ultimately restricts the mobility of the ossicular chain, particularly affecting the stapes and impairing sound transmission to the inner ear [2–4]. Although the etiopathogenesis of otosclerosis remains unclear, genetic and hereditary factors have been implicated, along with environmental influences such as measles virus infection and hormonal changes—including pregnancy, puberty, and menopause—that may trigger or exacerbate its symptoms [1, 5–7]. Otosclerosis is a prevalent condition, primarily affecting Caucasian women aged 20–40 years, with a reported prevalence of up to 0.4% [1, 8].

The most common clinical manifestation is progressive conductive hearing loss, which is also the primary reason for medical consultation and is frequently accompanied by tinnitus in approximately 50% of cases [2]. Vertigo affects 10% of patients [8], but its presence requires assessing other potential diagnoses, such as an enlarged vestibular aqueduct or third-window pathologies, with superior semicircular canal dehiscence being the most frequent.

The treatment includes both pharmacological and surgical options, with surgery being the preferred choice in most cases [9]. Thus, making an accurate diagnosis is essential to prevent unnecessary procedures and reduce the risk of complications [9, 10].

**Fig. 1:**
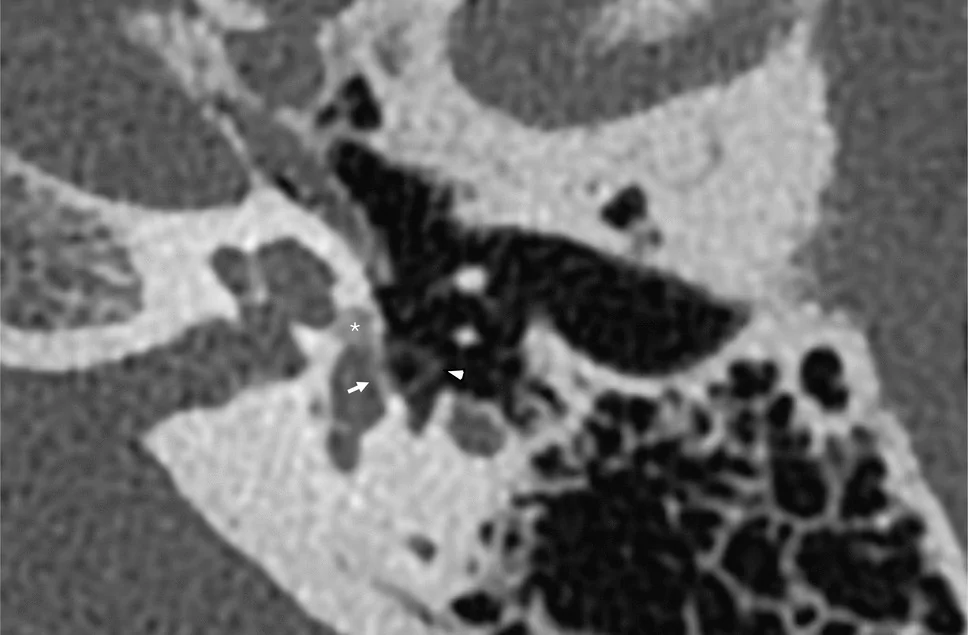
High-resolution computed tomography of the temporal bone. High-resolution computed tomography scan of the left temporal bone from a patient with otosclerosis included in this study. A demineralization focus is visible just anterior to the oval window, located in the bone between the cochlea and vestibule—known as the antefenestral region (*). The other two segmented structures in this study can also be seen: the stapes (arrowhead) and the oval window (arrow)

Currently, the diagnosis of otosclerosis continues to be primarily clinical, based on medical history, physical examination, and auditory assessments such as tuning fork tests, pure-tone audiometry, speech audiometry, and impedance testing [7]. In the past, a clinical suspicion alone was often sufficient to justify an exploratory tympanotomy to confirm stapes fixation by manipulating the ossicular chain or to directly visualize otosclerotic foci under a microscope. However, advancements in imaging techniques have increasingly supported the use of high-resolution computed tomography (HRCT) of the temporal bone to confirm the diagnosis. This allows for the identification of spongiotic or sclerotic bone foci around the stapes footplate, cochlea, and labyrinth [9, 10] (Fig. 1). The location of these otosclerotic foci determines the classification of the disease as fenestral or retrofenestral and correlates with the patient's audiovestibular symptoms [11].

Several studies have highlighted the critical role of this imaging modality in diagnosing otosclerosis [9, 10, 12]. Despite the potential for diagnostic errors—where nonspecific pericochlear signs, small vessels, or connective tissue around the stapes footplate and oval window have been misinterpreted as otosclerosis [13]—it has demonstrated a very high positive predictive value, ranging from 92 to 100%, with a specificity of approximately 95% [14].

However, despite advancements in the imaging technique and increasingly thinner slices aimed at detecting submillimetric and superficial foci, the sensitivity of HRCT remains limited [11]. At times, patients with otosclerosis may not exhibit detectable findings on temporal bone HRCT, even when evaluated by experienced radiologists. Additionally, like other imaging modalities, HRCT interpretation is operator-dependent, which can contribute to potential diagnostic errors. Previous studies have shown that radiological error rates can reach or exceed 30%, making otosclerosis a frequently underdiagnosed condition on HRCT in clinical practice [15, 16]. In these cases, definitive diagnosis can only be achieved intraoperatively.

This challenge highlights the need for new diagnostic strategies to enhance the accuracy of temporal bone HRCT while reducing its false-negative rate (FNR). The aim is to improve surgical decision-making and diagnostic accuracy, minimize unnecessary surgeries, and mitigate the potential complications associated with these procedures. In this context, radiomics—an emerging discipline focused on identifying imaging biomarkers [17]—offers a promising approach. Radiomics is based on the principle that medical images contain far more information about the pathophysiological processes of diseases than what the human eye can perceive [18]. These imaging biomarkers enable the development of Artificial Intelligence models, such as Machine Learning (ML) algorithms, capable of delivering highly precise and quantitative diagnostic assessments, paving the way for more reliable and personalized clinical decision-making [19].

## Material and Methods

### Patient Recruitment

The study sample comprised both cases and controls. Patient recruitment was conducted following the approval of our institution's Ethics Committee. All patients were enrolled from the same tertiary-level hospital in Spain.

The cases included all patients in our hospital with a surgically confirmed diagnosis of otosclerosis between 2021 and 2023 who also had a preoperative temporal bone HRCT. A total of 48 cases were included (25 females and 23 males), with a mean age of 53 ± 12 years. Of the included cases, 7 showed no signs of otosclerosis on the preoperative HRCT. The right ear was affected in 26 cases, while the left ear was affected in 23 cases (Table 1).

**Table 1:** Descriptive statistics of categorical variables

Variable	Non-Otosclerosis (*n* = 51)		Otosclerosis (*n* = 48)	
	*n*	%	*n*	b
Sex				
Female	16	31.37	25	52.08
Male	35	68.63	23	47.92
Ear				
Right	24	47.06	26	54.17
Left	27	52.94	22	45.83
HRCT findings				
Normal ear	51	100.00	7	14.58
Fenestral otosclerosis	0	0	39	81.25
Fenestral + retrofenestral otosclerosis	0	0	2	4.17

*HRCT * High-resolution computed tomography

A balanced sample of 51 controls was recruited (16 females and 35 males), with a mean age of 51 ± 17 years. The control group consisted of patients in whom otosclerosis had been clinically ruled out and who had undergone a temporal bone HRCT scan confirming normal middle and inner ear structures. All patients in our hospital who met these conditions during the period 2018–2023 were included. The affected ear was the right ear in 24 cases and the left ear in 27 cases (Table 1).

### Radiomic Feature Extraction and Selection

**Fig. 2:**
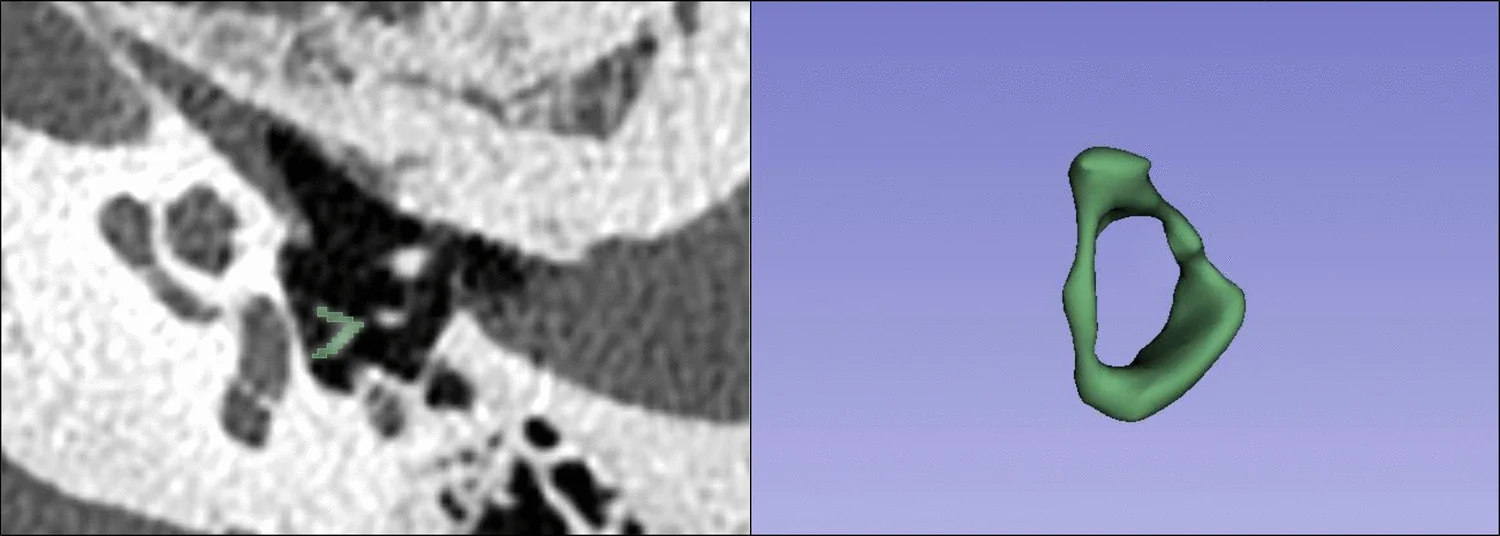
Stapes segmentation. Example of stapes segmentation from one of the patients included in this study (left) with its reconstructed three-dimensional volume (right)

HRCT images were acquired using a Siemens scanner (Siemens Healthineers, Erlangen, Germany) with a slice thickness of 0.4 mm and multiplanar reconstruction in a bone window for otosclerosis assessment. Three key structures (Fig. 1)—the stapes (Fig. 2), antefenestral region (AF), and oval window (OW)—were segmented using 3D Slicer v5.5.0 [20] by a radiologist (> 6 years of experience) and an ENT otologist, with all cases reviewed and agreed upon by both specialists.

Subsequently, radiomic feature extraction was carried out using the open-source package pyradiomics v3.1.0 [21], in accordance with the Image Biomarker Standardization Initiative standards [22]. For normalization and preprocessing, B-Spline interpolation was applied with a resampled voxel spacing of [1, 1, 1] mm to ensure isotropic voxels. A voxel array shift of 1000 was introduced to prevent negative values in certain radiomic extractions. Additionally, segmentation masks were validated by enforcing a minimum region of interest size of 1 voxel, ensuring accurate feature extraction.

From each segmented area, a total of 2016 radiomic features were extracted (6048 per image), including shape, first-order statistics, and texture features such as the Gray-Level Co-occurrence Matrix (GLCM), Gray-Level Run Length Matrix (GLRLM), Gray-Level Size Zone Matrix (GLSZM), Gray-Level Dependence Matrix (GLDM), and Neighboring Gray Tone Difference Matrix (NGTDM). Radiomic features were extracted from both the original image and after applying various transformations, including Wavelet, Logarithm, Square, Square Root, Exponential, and Laplacian of Gaussian, each with its respective adjusted bin width. A detailed description of the extracted features is available in Griethuysen et al. [23], while extraction settings are provided in Supplementary File S1.

Following the extraction process, a feature selection step was conducted to remove low-variance features and those with a Pearson correlation coefficient of 0.9 or higher. This process reduced the total number of features to 1317, comprising 456 from the segmentations of the stapes, 478 from the AF, and 385 from the OW.

### Statistical Analysis

A statistical description of the study variables was performed. Quantitative variables (age and radiomic features) were summarized using the mean ± standard deviation (SD) and median ± interquartile range (IQR); due to the extensive number of quantitative variables, descriptive statistics have not been included in this article but are available in the project repository [24]. Categorical variables (sex, CT findings, and ear) were described using absolute frequencies (*n*) and relative frequencies (%) (Table 1).

An inferential analysis was performed to identify significant differences in radiomic features between cases (otosclerosis) and controls (non-otosclerosis). Normality was assessed using the Shapiro–Wilk test and quantile–quantile plots. Student’s t-test or the Mann–Whitney U test was performed based on the distribution. Significance levels were adjusted for multiple testing using the Benjamin-Hochberg method to control the false discovery rate, with statistical significance set at α = 0.05.

### Machine Learning

Several ML algorithms were trained to develop a radiomic signature for the quantitative diagnosis of otosclerosis based on imaging findings. These included Support Vector Machine (SVM), K-Nearest Neighbors (KNN), Decision Tree (DT), Random Forest (RF), L2-regularized Logistic Regression (LR), Extreme Gradient Boosting (XGBoost) and Light Gradient Boosting Machine (LightGBM).

The models were trained using tenfold stratified cross-validation. *GridSearch* was used to optimize the hyperparameters of each algorithm (Supplementary File S2). Model performance was evaluated using the Area Under the Curve (AUC) of the Receiver Operating Characteristic (ROC) curve. Bootstrapping was used to estimate the SD and 95% confidence intervals (CI). FPR and precision-recall curve were calculated for the best model.

To prevent overfitting due to the high feature-to-sample ratio, various dimensionality reduction techniques outlined in Table 2 were applied, resulting in four feature subsets (Table 2). Each algorithm was then trained on all four subsets, with standardization applied beforehand. The subset that yielded the best-performing model was subsequently used for hierarchical clustering (HC).

**Table 2:** Feature subsets obtained from the application of different dimensionality reduction techniques

Subset	Dimensionality reduction technique	Total features
Subset 1—*Significant*	The 10 most significant features from HT, plus age and sex*	12
Subset 2 – *RF features*	The 10 most important features for a RF model, plus age and sex*	12
Subset 3 – *Significant* + *RF*	Common features between the 20 most significant from HT and the 20 most important for a RF, plus age and sex*	13
Subset 4 – PCA features	PCA components retaining 80% variance from the 40 most significant HT features, 40 most important RF features, plus age and sex*	14

*HT * Hypothesis testing, *RF * Random Forest,* PCA*  Principal component analysis

*Age and sex were included for their potential role as covariates

The top 10 features identified by HT are reported in Supplementary File S3, and those identified by RF in Supplementary File S5

### Programming Resources

The methodology of this study was implemented in Python v3.12.4 [25], using the pyradiomics v3.1.0 library [21] for radiomic feature extraction, scipy v1.14.1 [26] and statsmodels v0.14.4 [27] for statistical analysis, and scikit-learn v1.5.2 [28], xgboost v3.1.2 [29] and lightgbm v4.6.0 [30] for ML model training. In adherence to open science practices, all code from this project has been shared in a GitHub public repository [24].

## Results

### Radiomic Biomarkers

A total of 67 radiomic features showed significant differences between cases and controls: 11 from the stapes, 56 from the AF, and none from the OW. Significant statistical test results are in Supplementary File S3 and S4, with full results for all 1317 features available in the project repository [24].

**Fig. 3:**
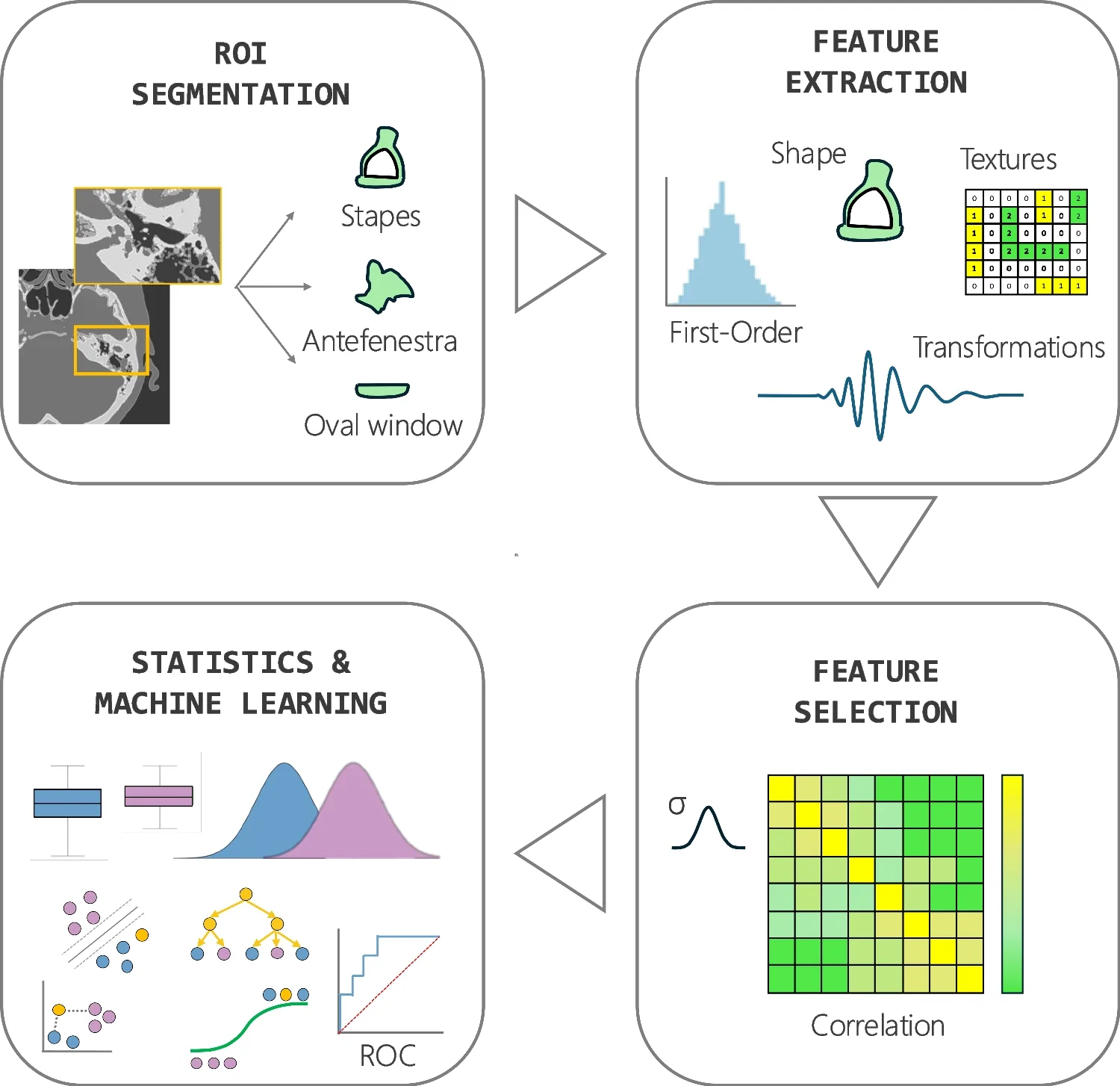
Methodology developed in the present study

In the stapes, applying a logarithmic transformation caused first-order features to exhibit statistically significant differences between otosclerosis cases and controls, which were not observed in the original HRCT images. In other words, by enhancing subtle density variations, the logarithmic transformation made the differences in the distributions of pixel values between the stapes of otosclerosis cases and controls more pronounced (Fig. 3).

**Fig. 4:**
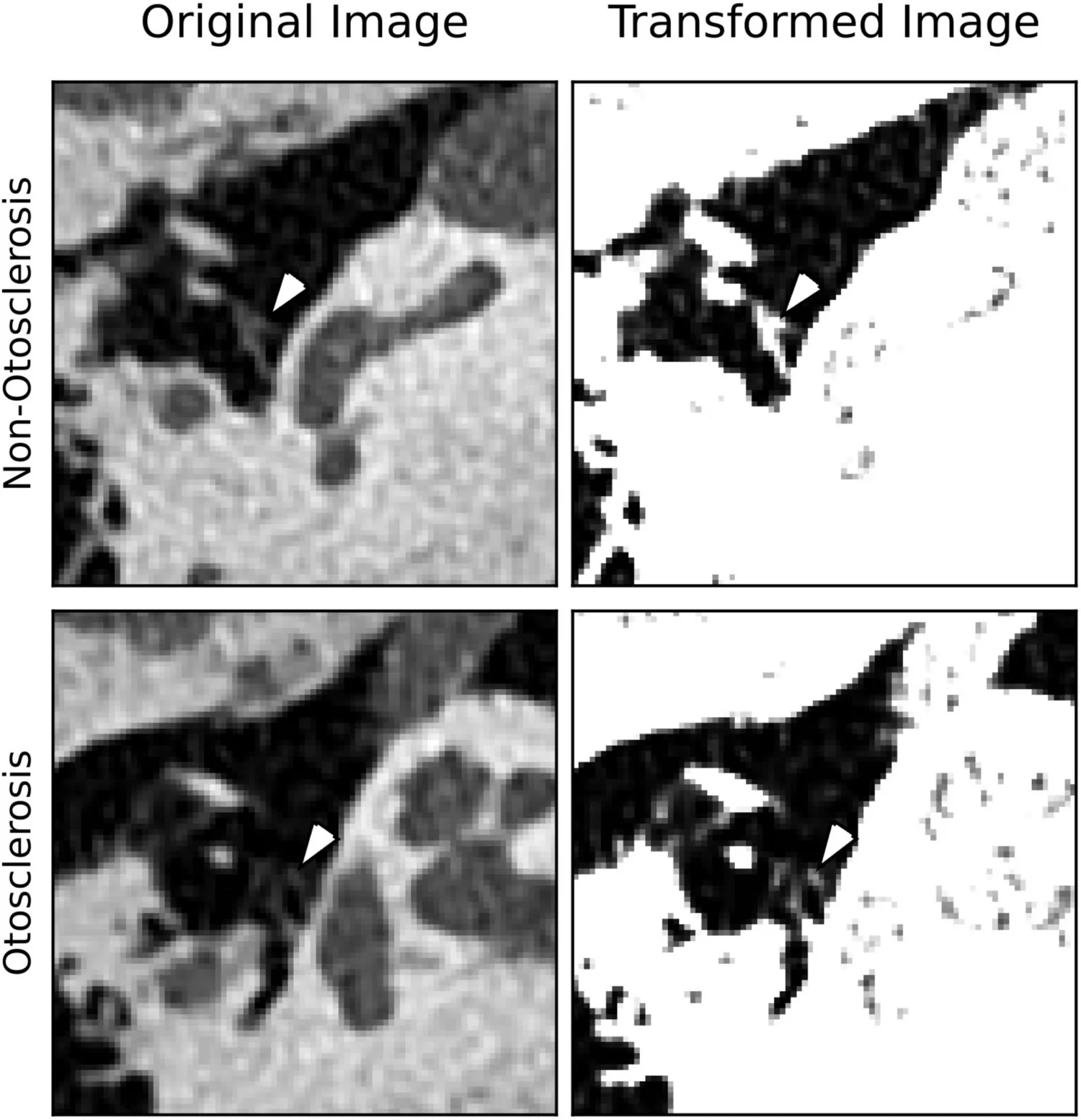
Results of the exponential imaging transformation on the stapes. High-resolution computed tomography images of the stapes (arrowhead) from a patient without otosclerosis (top) and with otosclerosis (bottom). Original images (left) and their exponential transformation (right) enhance stapes visibility and subtle pixel variations, highlighting heterogeneity. This transformation reveals a clear distinction: the stapes in the healthy patient appears homogeneous, whereas the stapes in the otosclerosis patient exhibits greater heterogeneity, likely due to otospongiosis. Additionally, after applying the transformation, GLSZM Large Area Emphasis (LAE) became significant, demonstrating that stapes affected by otosclerosis are more heterogeneous, as indicated by lower LAE values

Similarly, the exponential transformation showed that GLSZM Large Area Emphasis (LAE) was lower in otosclerosis cases (1.07 ± 0.22) than in controls (1.33 ± 0.49), indicating that the stapes in otosclerosis patients is more heterogeneous, with fewer large, uniform regions (Fig. 4). Additionally, the major axis of the stapes was significantly shorter in otosclerosis patients (3.86 ± 0.93 mm) compared to controls (4.60 ± 0.92 mm), highlighting structural differences associated with the disease.

In the AF region, shape remained unchanged, but attenuation was lower and texture was more heterogeneous in otosclerosis patients. Remarkably, of the 33 statistically significant features identified in the AF, only 6 showed statistically significant differences in the original image, whereas the remaining features became detectable only after applying transformations, underscoring the crucial role of these transformations in revealing subtle variations.

### Radiomic Signature

**Fig. 5:**
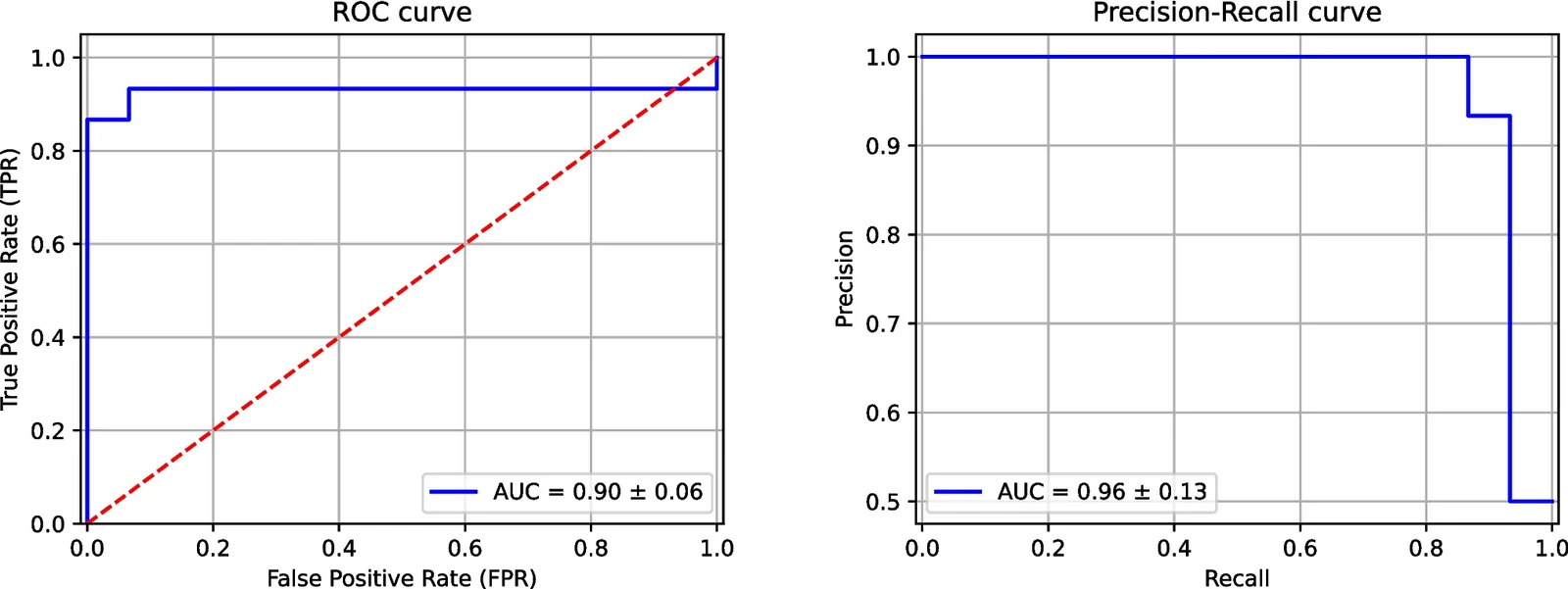
ROC and Precision-recall curves for the best-performing model—the L2-regularized logistic regression

The results of the ML algorithms training are presented in Table 3. The best model was the L2-regularized LR trained on the 10 most significant features plus age, and sex, achieving an AUC of 0.90 ± 0.06 and an FNR of 13.33% (Fig. 5). The LR formula is detailed in Supplementary File S6.

**Table 3:** Performance of machine learning models trained on different feature subsets (AUC ± SD [95% CI])

Model	Subset 1—*Significant*	Subset 2—*RF features*	Subset 3—*Significant* + *RF*	Subset 4—*PCA features*
SVM	0.85 ± 0.09 [0.62, 1.00]	0.60 ± 0.12 [0.37, 0.83]	0.80 ± 0.10 [0.60, 0.95]	0.75 ± 0.13 [0.48, 1.00]
K-NN	0.85 ± 0.08 [0.70, 1.00]	0.80 ± 0.10 [0.56, 0.96]	0.80 ± 0.10 [0.60, 0.96]	0.75 ± 0.13 [0.50, 1.00]
DT	0.65 ± 0.12 [0.42, 0.88]	0.75 ± 0.11 [0.51, 0.94]	0.70 ± 0.11 [0.47, 0.91]	0.75 ± 0.13 [0.49, 1.00]
RF	0.60 ± 0.12 [0.37, 0.82]	0.80 ± 0.10 [0.58, 1.00]	0.80 ± 0.10 [0.58, 0.96]	0.75 ± 0.11 [0.50, 1.00]
LR	**0.90 ± 0.06 [0.78, 1.00]**	0.77 ± 0.09 [0.60, 0.92]	0.80 ± 0.09 [0.62, 0.96]	0.83 ± 0.09 [0.66, 1.00]
XGBoost	0.70 ± 0.12 [0.44, 0.94]	0.70 ± 0.11 [0.45, 0.92]	0.75 ± 0.11 [0.50, 0.94]	0.81 ± 0.12 [0.56, 1.00]
LightGBM	0.75 ± 0.11 [0.52, 0.94]	0.70 ± 0.11 [0.47, 0.91]	0.75 ± 0.11 [0.51, 0.95]	0.81 ± 0.12 [0.56, 1.00]

*AUC* Area Under the Receiver Operating Characteristic Curve, *SD* Standard Deviation, *CI* Confidence Interval, *SVM* Support Vector Machine, *KNN* K-Nearest Neighbors, *DT* Decision Tree, *RF* Random Forest, *LR* L2-regularized Logistic Regression, *XGBoost *Extreme Gradient Boosting, *LightGBM *Light Gradient Boosting Machine

**Fig. 6:**
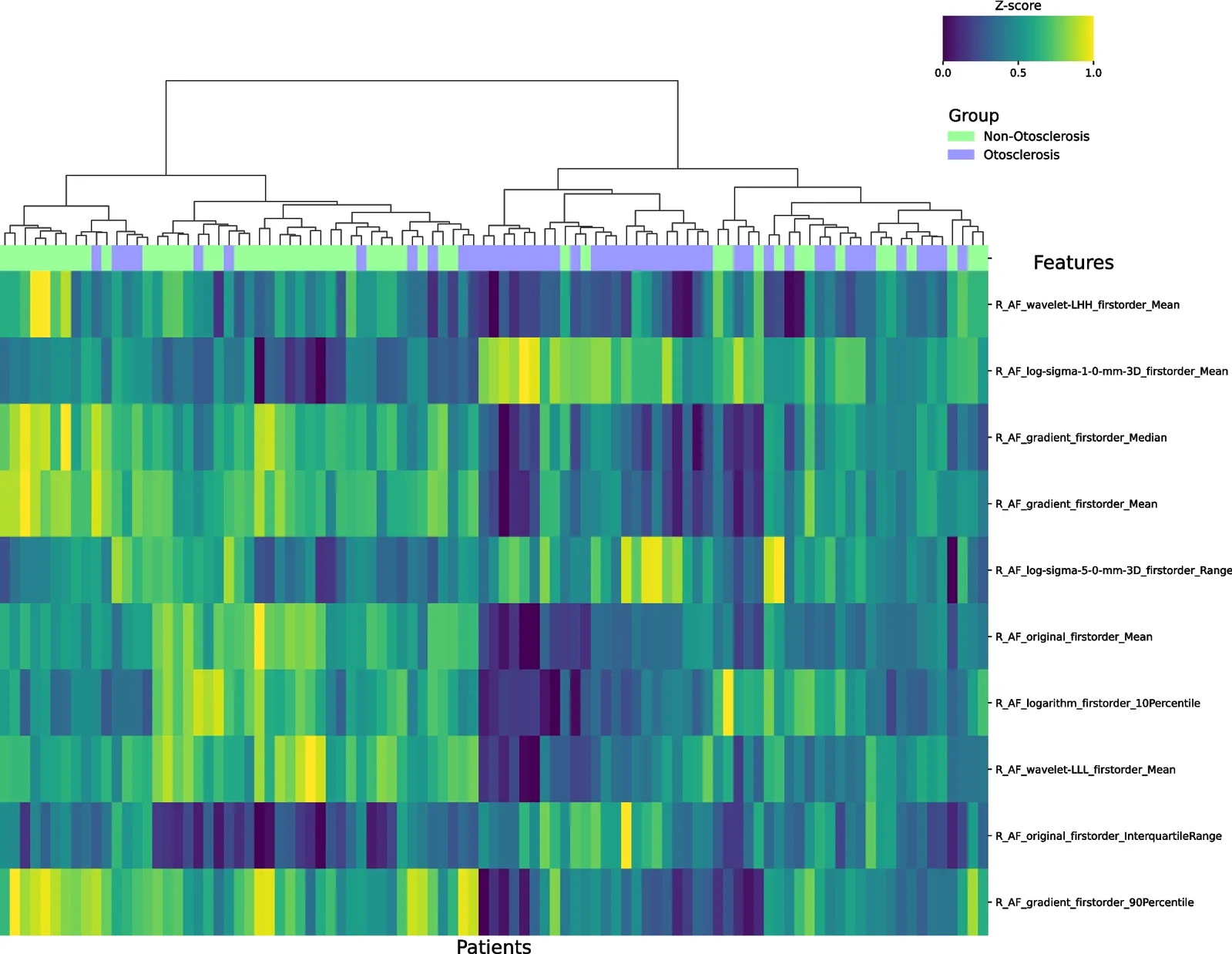
Hierarchical clustering of the ten most statistically significant features, after standardization

The HC of the ten most significant radiomic features revealed two distinct clusters: one with lower values, predominantly comprising otosclerosis patients, and another with higher values, mainly including control patients (Fig. 6).

## Discussion

Otosclerosis is a common cause of hearing loss in our population. The pathological process begins with increased vascularization and resorption of ear compact bone, followed by its replacement with immature, spongy bone. As the disease progresses, this bone gradually loses its vascularization, becoming sclerotic. This process, driven by the dysregulated activity of osteoblasts and osteoclasts, leads to disorganized bone deposition, stapes fixation, and subsequent impairment of sound transmission, ultimately resulting in progressive conductive or mixed hearing loss [2–4]. Treatment options include both medical and surgical management, being surgery the preferred option [9].

Diagnosis is primarily clinical, based on medical history and auditory assessments, but HRCT of the temporal bone has become an essential method for confirming otosclerosis [9]. HRCT can identify bone abnormalities around the stapes and cochlea, aiding disease classification. To date, several HRCT findings have been described, including decreased attenuation of the otic capsule due to demineralization and spongy bone formation in the otospongiotic phase [31]; changes in the thickness or contour of the otic capsule in the otosclerotic phase [31]; and increased thickness of the footplate [32].

Despite its high specificity, HRCT has limitations in sensitivity, with series reflecting high FNR, up to 45.4% [16]. It has been suggested that surgeries performed on patients with negative or inconclusive CT scans are associated with a higher risk of surgical complications [10]. This, along with the goal of better targeting surgical treatment, underscores the need for improved diagnostic methods.

Our study aims to conduct a quantitative analysis of high-resolution HRCT scans in patients with and without otosclerosis to identify radiomic imaging biomarkers that could enhance the accuracy, objectivity, and standardization of otosclerosis diagnosis. According to our literature review, only O'Toole Bom Braga et al. [33] have performed a similar quantitative analysis of ear structures. However, their study focused exclusively on the cochlea and was limited to a single temporal bone from a cadaver. In contrast, our study examines the stapes, the anterior focus (AF), and the oval window (OW) in 99 patients, 48 of whom had an intraoperative diagnosis of otosclerosis.

Our image analysis identified 67 radiomic biomarkers, highlighting the potential utility of radiomics in diagnosing otosclerosis. Among the three segmented regions, the AF exhibited the highest number of biomarkers (56 out of 67), which was expected given that this region has historically shown the most frequent radiological findings. The values of both shape and first-order features suggest increased heterogeneity and reduced attenuation in this region in patients with otosclerosis, without morphological alterations. These results correlate with previously reported radiological observations [31].

The remaining 11 of the 67 identified biomarkers were found in the stapes and included first-order, texture, and shape features. Patients with otosclerosis exhibited greater heterogeneity in the stapes, reflected in lower LAE values, likely due to bone remodeling processes. Additionally, a reduction in the major diameter of the stapes was observed, indicating not only textural but also morphological changes. This finding is consistent with previous studies reporting stapes deformation in otosclerosis due to a torsional effect caused by histological alterations [34]. As a result of this torsion, a shortening of the major diameter may occur, which we have observed for the first time in the literature. This effect was previously investigated by Anand et al. [34] by analyzing the distance between the horizontal segment of the facial nerve and the stapes head, as well as the angle formed between the stapes and the promontory. However, through three-dimensional stapes reconstruction and direct measurement of its major diameter, we have achieved a more precise quantification of this phenomenon.

No statistically significant differences were observed in the oval window, despite previous studies suggesting stapes footplate thickening [32]. This may be due to the small size of this anatomical region and the very subtle nature of the changes, which may exceed the resolution limits of standard imaging techniques, including HRCT. Future research using high-resolution imaging, such as Photon-Counting CT, which has demonstrated high diagnostic value in ossicular pathology [35], could provide further insights into this finding.

Of the 67 identified biomarkers, only eight were present in the original HRCT images, while 59 were revealed after applying image transformations. This suggests that image filtering techniques could enhance radiological interpretation by improving disease visibility. In our study, logarithmic transformations—designed to enhance low-attenuation areas such as otospongiotic bone— amplified previously reported differences in texture and attenuation between patients with and without otosclerosis in the stapes and AF region. Likewise, exponential transformations, which enhance subtle pixel intensity variations, further accentuated differences between cases and controls in the AF and allowed for the identification of a textural biomarker in the stapes, LAE, as illustrated in Fig. 4.

The identification of these biomarkers represents a significant step toward a more accurate and objective diagnosis of otosclerosis. To achieve this, various ML algorithms were trained, with L2-regularized LR demonstrating the best performance. This model achieved an AUC-ROC of 0.90 ± 0.06 (95% CI [0.78, 1.00]), exceeding previously reported accuracy for expert neuroradiologists and even more so for general radiologists [16]. Moreover, its FNR on the test set was 13.33%, lower than the 17.50% reported in the literature for neuroradiologists [16].

The ten most significant radiomic features were grouped using hierarchical clustering (HC), clearly distinguishing one cluster predominantly associated with control patients and another primarily linked to otosclerosis cases. These findings further strengthen the discriminative potential of these features.

These results highlight the potential of ML in developing radiomic signatures that can objectively quantify and standardize the diagnosis of otosclerosis while reducing missed diagnoses in imaging assessments. According to our literature review, this is the first radiomic study conducted on living patients with otosclerosis, focusing on the middle and inner ear. This highlights the originality and significance of our findings.

Future research could extend these methodologies to larger cohorts and different imaging modalities to validate and expand our observations, addressing the limitations of the present study. The purpose of this investigation was not to identify the single best-performing ML algorithm, but to provide proof-of-concept that integrating radiomics with ML can enhance the radiological diagnosis of otosclerosis by increasing precision and objectivity, thereby motivating further research in this field. First, the limited sample size restricts statistical power to detect meaningful differences between ML models, as reflected by the relatively wide 95% CIs. Second, external validation cohorts are needed to more rigorously evaluate reproducibility and generalizability across independent populations and imaging settings. Finally, future studies should consider incorporating data acquired with more sensitive imaging technologies, such as photon-counting CT, to determine whether higher-resolution and higher-contrast acquisitions enable extraction of more informative radiomic features from subtle anatomical structures, including the oval window.

## Conclusions

Our research demonstrates the potential of radiomic biomarkers to improve the diagnosis of otosclerosis using HRCT. The identification of 67 biomarkers, particularly in the stapes and AF region, highlights the usefulness of these techniques in detecting subtle structural changes that are not appreciable in conventional imaging. Furthermore, the use of image transformations has enhanced the visibility of bone alterations, suggesting their integration into clinical practice. The developed ML model, an L2-regularized LR, achieved an accuracy surpassing that reported in some series for expert neuroradiologists, reinforcing the feasibility of radiomic signatures as an objective and standardized diagnostic tool. While our findings represent a significant advancement, future research with larger cohorts and new imaging modalities, such as Photon-Counting CT, will be essential to validate and expand their applicability in clinical practice.

## Supplementary Information

Below is the link to the electronic supplementary material.

ESM 1 (DOCX 493 KB)

## Data Availability

The data supporting the findings of this study are not publicly available as they involve patient data subject to ethical and legal restrictions.
