# Supplementary material for: A New Insight into Imaging Diagnosis of Otosclerosis Enhanced by Machine Learning and Radiomics
Source: J Imaging Inform Med. 2026 Jan 30;39(5):4472–81. doi: 10.1007/s10278-026-01843-0 (PMC13694669; doi:10.1007/s10278-026-01843-0)
Supplement: Supplementary file 1 [file 10278_2026_1843_MOESM1_ESM.docx]

**A NEW INSIGHT INTO IMAGING DIAGNOSIS OF OTOSCLEROSIS ENHANCED BY MACHINE LEARNING AND RADIOMICS**

SUPPLEMENTARY MATERIAL

**Supplementary S1. Radiomic feature extraction settings.** A total of 6048 radiomic features were extracted from each image—2016 features per segmentation—using pyradiomics v3.1.0. The extraction process was performed with the parameters specified below. The *ImageType* section details the filters applied to the different radiomic feature categories listed under *featureClass*. For a more comprehensive explanation of these parameters, please refer to the pyradiomics documentation.

imageType:

Original:

binWidth: 25.010000 (Stapes), 35.930000 (AF), 18.300000 (OW)

Square:

binWidth: 6.459188, (Stapes), 39.234190 (AF), 10.856929 (OW)

SquareRoot:

binWidth: 48.464846, (Stapes), 26.650614 (AF), 20.135843 (OW)

Logarithm:

binWidth: 79.474406, (Stapes), 10.514937 (AF), 10.346025 (OW)

Exponential:

binWidth: 0.332049, (Stapes), 23.866770 (AF), 0.792184 (OW)

Gradient:

binWidth: 23.510929, (Stapes), 29.831300 (AF), 14.756986 (OW)

LoG:

binWidth: 9.0 (Stapes), 15.0 (AF), 5.2 (OW)

sigma: [1.0, 2.0, 3.0, 4.0, 5.0]

Wavelet:

binWidth: 25, (Stapes), 52 (AF), 15 (OW)

LBP3D:

binWidth: 1.0

featureClass:

shape:

firstorder:

glcm:

- 'Autocorrelation'

- 'JointAverage'

- 'ClusterProminence'

- 'ClusterShade'

- 'ClusterTendency'

- 'Contrast'

- 'Correlation'

- 'DifferenceAverage'

- 'DifferenceEntropy'

- 'DifferenceVariance'

- 'JointEnergy'

- 'JointEntropy'

- 'Imc1'

- 'Imc2'

- 'Idm'

- 'Idmn'

- 'Id'

- 'Idn'

- 'InverseVariance'

- 'MaximumProbability'

- 'SumEntropy'

- 'SumSquares'

glrlm:

glszm:

gldm:

ngtdm:

setting:

interpolator: 'sitkBSpline'

resampledPixelSpacing: [1, 1, 1]

padDistance: 10

minimumROIDimensions: 1

minimumROISize: 1

correctMask: true

voxelArrayShift: 1000

label: 1

**Supplementary S2. GridSearch used for training Machine Learning models.** Several Machine Learning algorithms were trained using the subsets described in Table II. These models were trained using 10-fold stratified cross-validation and GridSearch to optimize the hyperparameters of each algorithm. The trained algorithms were: Support Vector Machine (SVM), K-Nearest Neighbors (KNN), Decision Tree (DT), Random Forest (RF), L2-regularized Logistic Regression (LR), Extreme Gradient Boosting (XGBoost) and Light Gradient Boosting Machine (LightGBM).

svm_param_grid = {'C': [0.4, 0.6, 0.8, 1.0, 1.2, 1.4, 1.6, 1.8],

‘gamma': ['scale', 1, 0.1, 0.01, 0.001],

'kernel': ['linear', 'poly', 'rbf', 'sigmoid']}

param_grid_knn = {'n_neighbors': [3, 5, 7, 9, 11],

'weights': ['uniform', 'distance'],

'metric': ['euclidean', 'manhattan', 'minkowski'],

'algorithm': ['auto', 'ball_tree', 'kd_tree', 'brute'],

'leaf_size': [20, 30, 40, 50],

'p': [1, 2]}

param_grid_dt = {'criterion': ['gini', 'entropy'],

'splitter': ['best', 'random'],

'max_depth': [2, 4, 5, 6, 8, 10],

'min_samples_split': [2, 5, 6, 8],

'min_samples_leaf': [2, 4, 6],

'max_features': ['sqrt', 'log2']}

param_grid_rf = {'n_estimators': [50, 100],

'max_depth': [None, 2, 4, 10],

'min_samples_split': [2, 3, 6],

'min_samples_leaf': [1, 2, 4]}

param_grid_lr = {'C': [n/100 for n in range(1,200)]}

param_grid_xgb = {'n_estimators': [100, 300],

'learning_rate': [0.05, 0.1],

'max_depth': [2, 3, 4],

'subsample': [0.8, 1.0],

'colsample_bytree': [0.8, 1.0],

'reg_lambda': [1, 5],

'min_child_weight': [1, 5]}

param_grid_lgbm = {'n_estimators': [100, 300],

'learning_rate': [0.05, 0.1],

'max_depth': [3, 5],

'num_leaves': [7, 15, 31],

'subsample': [0.8, 1.0],

'subsample_freq': [1],

'colsample_bytree': [0.8, 1.0],

'reg_lambda': [0, 1, 5],

'min_child_samples': [5, 10, 20]}

**Supplementary S3. Statistically significant test results for radiomic features.** 67 radiomic features showed significant differences between cases and controls: 11 from the stapes, 56 from the AF, and none from the OW. Below are the results of the significant tests, including Student's t-test for normal variables (Table S3A) and the Mann-Whitney U test for non-normal variables (Table S3B). The most statistically significant features of the stapes and antefenestral (AF) region are shown in Figure S3.

| Table S3A. **Significant Results from Student's T-Tests for Normally Distributed Variables.** | | | | | |
| --- | --- | --- | --- | --- | --- |
| **Variable** | **X̄ ± SD** | **Non-otosclerosis**  **(X̄ ± SD)** | **Otosclerosis**  **(X̄ ± SD)** | **p-value** | **BH corrected p-value** |
| wavelet-LHH_firstorder_Mean | 26.56 ± 59.98 | 54.06 ± 57.64 | -2.67 ± 47.69 | <0.0001 | 0.0002 |
| log-sigma-1-0-mm-3D_firstorder_Mean | -476.73 ± 105.01 | -520.45 ± 95.66 | -430.27 ± 94.77 | <0.0001 | 0.0012 |
| gradient_firstorder_Median | 1002.43 ± 129.67 | 1054.04 ± 114.66 | 947.60 ± 122.95 | <0.0001 | 0.0017 |
| gradient_firstorder_Mean | 993.82 ± 106.42 | 1037.02 ± 83.86 | 947.92 ± 109.34 | <0.0001 | 0.0017 |
| log-sigma-5-0-mm-3D_firstorder_Range | 604.59 ± 103.70 | 564.45 ± 90.82 | 647.25 ± 100.21 | <0.0001 | 0.0017 |
| original_firstorder_Mean | 1588.71 ± 210.71 | 1670.18 ± 196.39 | 1502.14 ± 191.54 | <0.0001 | 0.0017 |
| logarithm_firstorder_10Percentile | 2220.44 ± 150.23 | 2279.52 ± 132.99 | 2157.66 ± 142.93 | <0.0001 | 0.0017 |
| wavelet-LLL_firstorder_Mean | 3486.05 ± 432.43 | 3647.99 ± 371.85 | 3313.98 ± 429.08 | 0.0001 | 0.0023 |
| original_firstorder_InterquartileRange | 604.07 ± 220.58 | 521.59 ± 198.12 | 691.70 ± 210.89 | 0.0001 | 0.0023 |
| gradient_firstorder_90Percentile | 1459.86 ± 187.04 | 1530.19 ± 159.84 | 1385.13 ± 186.26 | 0.0001 | 0.0023 |
| original_firstorder_Skewness | -0.66 ± 0.51 | -0.85 ± 0.44 | -0.46 ± 0.51 | 0.0001 | 0.0027 |
| lbp-3D-m1_firstorder_MeanAbsoluteDeviation | 3.42 ± 0.59 | 3.21 ± 0.54 | 3.65 ± 0.55 | 0.0001 | 0.0032 |
| lbp-3D-m1_glcm_Contrast | 35.91 ± 12.68 | 31.38 ± 10.87 | 40.73 ± 12.79 | 0.0002 | 0.0040 |
| log-sigma-5-0-mm-3D_firstorder_InterquartileRange | 242.73 ± 58.88 | 222.35 ± 56.35 | 264.39 ± 54.04 | 0.0003 | 0.0055 |
| log-sigma-4-0-mm-3D_firstorder_Range | 732.45 ± 112.57 | 693.85 ± 106.54 | 773.46 ± 104.93 | 0.0003 | 0.0056 |
| log-sigma-4-0-mm-3D_firstorder_Entropy | 4.26 ± 0.34 | 4.14 ± 0.33 | 4.38 ± 0.31 | 0.0003 | 0.0056 |
| STAPES_original_shape_MajorAxisLength | 4.23 ± 0.99 | 4.60 ± 0.92 | 3.86 ± 0.93 | 0.0003 | 0.0056 |
| wavelet-LLL_firstorder_10Percentile | 2109.69 ± 565.24 | 2301.78 ± 530.90 | 1905.60 ± 532.69 | 0.0004 | 0.0057 |
| log-sigma-2-0-mm-3D_firstorder_90Percentile | 75.14 ± 121.04 | 34.29 ± 118.56 | 118.55 ± 108.90 | 0.0004 | 0.0058 |
| lbp-3D-m1_firstorder_90Percentile | 11.92 ± 1.74 | 11.34 ± 1.69 | 12.54 ± 1.59 | 0.0005 | 0.0067 |
| log-sigma-1-0-mm-3D_firstorder_Minimum | -971.57 ± 140.61 | -1017.59 ± 127.71 | -922.68 ± 138.32 | 0.0006 | 0.0086 |
| lbp-3D-m1_firstorder_InterquartileRange | 5.76 ± 1.48 | 5.29 ± 1.38 | 6.27 ± 1.42 | 0.0008 | 0.0100 |
| log-sigma-2-0-mm-3D_firstorder_Mean | -189.46 ± 92.00 | -218.23 ± 90.67 | -158.89 ± 83.98 | 0.0010 | 0.0133 |
| log-sigma-4-0-mm-3D_firstorder_InterquartileRange | 293.84 ± 65.76 | 273.48 ± 67.66 | 315.48 ± 56.73 | 0.0011 | 0.0139 |
| original_firstorder_MeanAbsoluteDeviation | 377.68 ± 95.67 | 348.42 ± 97.24 | 408.76 ± 84.33 | 0.0013 | 0.0151 |
| wavelet-LLH_glszm_GrayLevelNonUniformity | 1.32 ± 0.17 | 1.26 ± 0.15 | 1.37 ± 0.17 | 0.0013 | 0.0151 |
| lbp-3D-m1_firstorder_Maximum | 15.67 ± 2.47 | 14.93 ± 2.51 | 16.47 ± 2.19 | 0.0015 | 0.0163 |
| logarithm_firstorder_Mean | 2365.15 ± 131.32 | 2404.64 ± 134.85 | 2323.20 ± 114.55 | 0.0016 | 0.0169 |
| STAPES_log-sigma-3-0-mm-3D_firstorder_InterquartileRange | 247.74 ± 137.09 | 291.81 ± 140.10 | 202.68 ± 119.43 | 0.0017 | 0.0176 |
| STAPES_log-sigma-2-0-mm-3D_firstorder_MeanAbsoluteDeviation | 161.52 ± 67.93 | 182.59 ± 65.77 | 139.97 ± 63.85 | 0.0026 | 0.0253 |
| STAPES_log-sigma-2-0-mm-3D_firstorder_Range | 476.36 ± 217.66 | 543.52 ± 202.69 | 407.68 ± 213.00 | 0.0028 | 0.0262 |
| wavelet-LLL_glcm_Correlation | 0.06 ± 0.17 | 0.01 ± 0.15 | 0.11 ± 0.18 | 0.0041 | 0.0374 |
| square_glcm_Autocorrelation | 668.92 ± 275.91 | 744.31 ± 280.40 | 588.83 ± 249.75 | 0.0044 | 0.0379 |
| lbp-3D-m1_glcm_Autocorrelation | 52.37 ± 12.13 | 49.04 ± 11.39 | 55.91 ± 12.00 | 0.0044 | 0.0379 |
| lbp-3D-m1_glrlm_HighGrayLevelRunEmphasis | 64.03 ± 14.31 | 60.11 ± 13.40 | 68.18 ± 14.20 | 0.0046 | 0.0384 |
| STAPES_log-sigma-2-0-mm-3D_firstorder_InterquartileRange | 204.62 ± 110.55 | 236.58 ± 116.21 | 171.95 ± 95.03 | 0.0051 | 0.0416 |
| squareroot_firstorder_90Percentile | 2311.03 ± 97.35 | 2336.83 ± 98.26 | 2283.61 ± 89.42 | 0.0058 | 0.0460 |
| BH = Benjamini-Hochberg | | | | | |

| Table S3B. **Significant Results from Mann-Whitney's U-Tests for Non-Normally Distributed Variables.** | | | | | | | | | | | | | | |  |
| --- | --- | --- | --- | --- | --- | --- | --- | --- | --- | --- | --- | --- | --- | --- | --- |
|  |  |  | **Non-otosclerosis** | |  | | **Otosclerosis** | | |  | |  |  |  | |
| **Variables** | **X̄ ± SD** | **M ± IQR** | **X̄ ± SD** | **M ± IQR** |  | **X̄ ± SD** | | **M ± IQR** | **p-value** | | **BH corrected p-value** | | | |  |
| AF_exponential_gldm_DependenceVariance | 1.29 ± 2.52 | 0.44 ± 1.03 | 0.47 ± 0.58 | 0.25 ± 0.36 |  | 2.15 ± 3.38 | | 1.13 ± 2.46 | <0.0001 | | 0.0046 | | | |  |
| AF_exponential_glrlm_LongRunLowGrayLevelEmphasis | 0.26 ± 0.18 | 0.21 ± 0.23 | 0.18 ± 0.12 | 0.17 ± 0.15 |  | 0.34 ± 0.21 | | 0.30 ± 0.24 | <0.0001 | | 0.0046 | | | |  |
| AF_wavelet-LHH_firstorder_RootMeanSquared | 1124.78 ± 79.11 | 1113.52 ± 92.55 | 1158.00 ± 80.56 | 1155.97 ± 82.61 |  | 1089.49 ± 60.61 | | 1087.66 ± 86.27 | <0.0001 | | 0.0059 | | | |  |
| AF_lbp-3D-m1_glcm_ClusterProminence | 2833.87 ± 2101.69 | 2251.73 ± 2530.32 | 2042.78 ± 1541.94 | 1655.52 ± 1719.29 |  | 3674.41 ± 2297.86 | | 3463.28 ± 2682.94 | 0.0001 | | 0.0106 | | | |  |
| AF_log-sigma-1-0-mm-3D_firstorder_Energy | 9959101.77 ± 4623689.72 | 9214831.37 ± 6051494.87 | 8193772.74 ± 3661914.82 | 7571286.98 ± 4305311.62 |  | 11834763.86 ± 4830769.57 | | 11525652.92 ± 4605084.63 | 0.0001 | | 0.0106 | | | |  |
| AF_exponential_glszm_LargeAreaEmphasis | 3.33 ± 4.20 | 1.91 ± 1.87 | 2.05 ± 1.15 | 1.56 ± 0.90 |  | 4.70 ± 5.63 | | 2.72 ± 3.06 | 0.0001 | | 0.0140 | | | |  |
| AF_log-sigma-5-0-mm-3D_glszm_LargeAreaHighGrayLevelEmphasis | 1229.52 ± 642.08 | 1101.54 ± 660.95 | 985.44 ± 374.32 | 908.06 ± 512.44 |  | 1488.86 ± 759.75 | | 1358.00 ± 847.76 | 0.0001 | | 0.0154 | | | |  |
| AF_original_firstorder_Kurtosis | 3.03 ± 1.15 | 2.73 ± 1.29 | 3.39 ± 1.23 | 2.99 ± 1.27 |  | 2.65 ± 0.94 | | 2.31 ± 0.85 | 0.0001 | | 0.0164 | | | |  |
| AF_log-sigma-4-0-mm-3D_glcm_Correlation | 0.49 ± 0.14 | 0.51 ± 0.17 | 0.45 ± 0.13 | 0.45 ± 0.19 |  | 0.54 ± 0.14 | | 0.56 ± 0.12 | 0.0002 | | 0.0177 | | | |  |
| AF_lbp-3D-m1_ngtdm_Complexity | 281.68 ± 123.34 | 270.92 ± 182.05 | 232.94 ± 92.88 | 218.97 ± 112.27 |  | 333.47 ± 131.24 | | 341.51 ± 208.47 | 0.0002 | | 0.0177 | | | |  |
| AF_exponential_glrlm_GrayLevelNonUniformity | 2.51 ± 1.31 | 2.12 ± 1.57 | 2.06 ± 0.91 | 1.82 ± 1.27 |  | 2.99 ± 1.50 | | 2.54 ± 1.80 | 0.0002 | | 0.0177 | | | |  |
| AF_log-sigma-4-0-mm-3D_glcm_ClusterProminence | 477055.91 ± 390665.34 | 358769.32 ± 390528.37 | 373341.18 ± 356254.12 | 259971.98 ± 253970.09 |  | 587252.82 ± 398918.13 | | 434712.27 ± 505818.10 | 0.0002 | | 0.0214 | | | |  |
| AF_log-sigma-4-0-mm-3D_ngtdm_Complexity | 1913.20 ± 605.22 | 1905.41 ± 768.56 | 1718.12 ± 553.59 | 1612.74 ± 623.03 |  | 2120.48 ± 593.76 | | 2058.89 ± 524.60 | 0.0003 | | 0.0214 | | | |  |
| AF_exponential_glszm_ZonePercentage | 0.77 ± 0.14 | 0.80 ± 0.20 | 0.82 ± 0.11 | 0.86 ± 0.14 |  | 0.72 ± 0.15 | | 0.73 ± 0.21 | 0.0003 | | 0.0237 | | | |  |
| STAPES_original_shape_Maximum3DDiameter | 3.52 ± 0.68 | 3.32 ± 0.96 | 3.77 ± 0.61 | 3.74 ± 0.93 |  | 3.27 ± 0.67 | | 3.32 ± 0.52 | 0.0004 | | 0.0237 | | | |  |
| AF_original_firstorder_10Percentile | 960.48 ± 339.93 | 887.80 ± 473.80 | 1081.40 ± 351.72 | 1073.80 ± 602.00 |  | 832.00 ± 276.78 | | 796.00 ± 399.68 | 0.0004 | | 0.0237 | | | |  |
| STAPES_log-sigma-3-0-mm-3D_firstorder_Range | 535.87 ± 243.03 | 576.67 ± 303.64 | 618.24 ± 229.61 | 693.75 ± 271.18 |  | 451.63 ± 229.28 | | 534.90 ± 352.94 | 0.0004 | | 0.0237 | | | |  |
| STAPES_log-sigma-2-0-mm-3D_firstorder_90Percentile | 615.69 ± 155.07 | 643.25 ± 219.10 | 662.55 ± 164.53 | 717.19 ± 192.07 |  | 567.76 ± 129.86 | | 584.39 ± 178.36 | 0.0004 | | 0.0237 | | | |  |
| STAPES_log-sigma-3-0-mm-3D_firstorder_90Percentile | 594.36 ± 229.01 | 619.63 ± 304.03 | 653.75 ± 244.25 | 713.45 ± 211.29 |  | 533.62 ± 197.02 | | 510.58 ± 229.95 | 0.0007 | | 0.0367 | | | |  |
| AF_square_firstorder_Skewness | -0.06 ± 0.52 | -0.14 ± 0.73 | -0.23 ± 0.43 | -0.28 ± 0.61 |  | 0.13 ± 0.55 | | 0.15 ± 0.69 | 0.0009 | | 0.0466 | | | |  |
| STAPES_log-sigma-3-0-mm-3D_firstorder_MeanAbsoluteDeviation | 186.30 ± 83.28 | 206.05 ± 114.38 | 213.13 ± 79.78 | 224.63 ± 85.14 |  | 158.87 ± 78.47 | | 168.90 ± 113.91 | 0.0010 | | 0.0466 | | | |  |
| AF_exponential_firstorder_10Percentile | 35.40 ± 49.06 | 13.95 ± 30.39 | 50.40 ± 62.33 | 26.48 ± 64.44 |  | 19.46 ± 19.50 | | 11.41 ± 17.06 | 0.0010 | | 0.0466 | | | |  |
| AF_squareroot_firstorder_Kurtosis | 4.37 ± 2.65 | 3.38 ± 2.75 | 4.82 ± 2.45 | 3.93 ± 2.99 |  | 3.90 ± 2.80 | | 2.83 ± 1.29 | 0.0014 | | 0.0471 | | | |  |
| AF_square_glcm_ClusterShade | -433.59 ± 2814.07 | -503.92 ± 2327.48 | -1199.82 ± 1946.04 | -613.02 ± 2057.11 |  | 380.54 ± 3342.03 | | 457.80 ± 3459.78 | 0.0011 | | 0.0471 | | | |  |
| AF_log-sigma-2-0-mm-3D_glcm_Autocorrelation | 349.37 ± 167.28 | 320.74 ± 189.90 | 299.10 ± 143.58 | 293.43 ± 150.50 |  | 402.79 ± 175.39 | | 378.05 ± 166.11 | 0.0012 | | 0.0471 | | | |  |
| AF_log-sigma-4-0-mm-3D_glcm_ClusterShade | -3848.06 ± 3593.32 | -2601.63 ± 4046.46 | -2909.58 ± 3344.51 | -1831.26 ± 3021.60 |  | -4845.20 ± 3612.73 | | -3828.46 ± 5605.04 | 0.0012 | | 0.0471 | | | |  |
| AF_wavelet-HLL_ngtdm_Contrast | 42.47 ± 40.60 | 29.64 ± 36.03 | 51.46 ± 44.39 | 39.61 ± 33.55 |  | 32.91 ± 34.04 | | 19.98 ± 22.01 | 0.0011 | | 0.0471 | | | |  |
| STAPES_exponential_glszm_LargeAreaEmphasis | 1.20 ± 0.40 | 1.00 ± 0.00 | 1.33 ± 0.49 | 1.00 ± 0.75 |  | 1.07 ± 0.22 | | 1.00 ± 0.00 | 0.0013 | | 0.0471 | | | |  |
| AF_lbp-3D-m1_glszm_ZoneEntropy | 3.72 ± 0.33 | 3.78 ± 0.41 | 3.62 ± 0.33 | 3.68 ± 0.48 |  | 3.83 ± 0.31 | | 3.85 ± 0.29 | 0.0013 | | 0.0471 | | | |  |
| AF_lbp-3D-k_glszm_GrayLevelVariance | 0.74 ± 0.36 | 0.69 ± 0.16 | 0.62 ± 0.20 | 0.67 ± 0.09 |  | 0.87 ± 0.44 | | 0.69 ± 0.59 | 0.0012 | | 0.0471 | | | |  |
| BH = Benjamini-Hochberg | | | | | | | | | | | | | | |  |

Non -

Non -

Non -

Non -

**Figure S3. Most statistically significant features of the stapes and antefenestral (AF) region.**

**Supplementary S4. Statistical comparison of baseline categorical variables between otosclerosis and control groups.** Baseline categorical variables were compared between patients with otosclerosis and control subjects. The variable *HRCT findings* was excluded from the analysis, as it is specific to otosclerosis and therefore not applicable to the control group.

Contingency tables were constructed for each variable, and the χ² statistic was used to evaluate independence between groups. Given the relatively small sample size, p-values were estimated using a Monte-Carlo permutation approach under the null hypothesis of independence (10,000 permutations). No correction for multiple comparisons was applied, as these analyses were intended to describe baseline characteristics rather than to support confirmatory inference (Table S4).

Statistical analysis revealed a statistically significant difference in sex distribution, with otosclerosis being more prevalent among women in our sample. This finding is consistent with the established epidemiology of the disease. Accordingly, sex was treated as a covariate and was included in all four subsets used to train the machine learning algorithms (Table II). The complete statistical analysis supporting these results is available in the project repository [25].

| Table S4. **Results of χ² Hypothesis Tests for Baseline Categorical Variables** | | | | | | | |
| --- | --- | --- | --- | --- | --- | --- | --- |
|  | **Non-Otosclerosis** | |  | **Otosclerosis** | |  |  |
| **Variable** | **n** | **%** |  | **n** | **%** |  | **p-value** |
|  |  |  |  |  |  |  |  |
| Sex |  |  |  |  |  |  |  |
| Female | 16 | 31,37 |  | 25 | 52,08 |  | 0.0436 |
| Male | 35 | 68,63 |  | 23 | 47,92 |  |  |
| Ear |  |  |  |  |  |  |  |
| Right | 24 | 47,06 |  | 26 | 54,17 |  | 0.5445 |
| Left | 27 | 52,94 |  | 22 | 45,83 |  |  |
|  |  |  |  |  |  |  |  |
|  | | | | | | | |

**Supplementary S5. Top 40 features selected by Random Forest.** The 40 most important features were selected using a Random Forest model trained with RandomForestClassifier (scikit-learn v1.5.2, n_estimators=100). Feature importance was assessed based on its impact on decision splits, ranking features by their contribution to predictive power. The top 40 were retained for further analysis, with details provided in Table S4.

| Table S5. **Top 40 features selected by Random Forest.** | | |
| --- | --- | --- |
| **Feature** | **Importance** |  |
| AF_logarithm_firstorder_10Percentile | 0.0204 |  |
| AF_wavelet-LHH_firstorder_RootMeanSquared | 0.0154 |  |
| AF_log-sigma-4-0-mm-3D_firstorder_Entropy | 0.0125 |  |
| AF_gradient_firstorder_Mean | 0.0118 |  |
| AF_original_firstorder_Mean | 0.0100 |  |
| AF_wavelet-LHH_glszm_LargeAreaEmphasis | 0.0088 |  |
| AF_lbp-3D-m1_glcm_Contrast | 0.0084 |  |
| AF_wavelet-LHH_firstorder_Mean | 0.0082 |  |
| AF_wavelet-HLL_ngtdm_Contrast | 0.0080 |  |
| AF_exponential_gldm_DependenceVariance | 0.0077 |  |
| AF_log-sigma-4-0-mm-3D_glcm_ClusterProminence | 0.0077 |  |
| AF_log-sigma-1-0-mm-3D_firstorder_Mean | 0.0069 |  |
| AF_log-sigma-2-0-mm-3D_firstorder_90Percentile | 0.0066 |  |
| AF_original_firstorder_Kurtosis | 0.0066 |  |
| AF_log-sigma-5-0-mm-3D_firstorder_InterquartileRange | 0.0066 |  |
| AF_log-sigma-4-0-mm-3D_firstorder_Range | 0.0063 |  |
| AF_squareroot_firstorder_Kurtosis | 0.0058 |  |
| AF_lbp-3D-m1_firstorder_MeanAbsoluteDeviation | 0.0056 |  |
| AF_lbp-3D-m1_glcm_ClusterProminence | 0.0055 |  |
| AF_square_glcm_ClusterProminence | 0.0054 |  |
| AF_log-sigma-4-0-mm-3D_glcm_ClusterShade | 0.0053 |  |
| AF_wavelet-HLL_firstorder_Maximum | 0.0052 |  |
| STAPES_log-sigma-1-0-mm-3D_firstorder_Energy | 0.0050 |  |
| AF_logarithm_firstorder_Mean | 0.0049 |  |
| AF_log-sigma-4-0-mm-3D_glszm_SizeZoneNonUniformity | 0.0049 |  |
| AF_square_firstorder_Skewness | 0.0049 |  |
| AF_lbp-3D-k_glcm_ClusterProminence | 0.0049 |  |
| AF_wavelet-HLL_ngtdm_Strength | 0.0046 |  |
| AF_lbp-3D-m1_ngtdm_Complexity | 0.0045 |  |
| OW_wavelet-LHH_firstorder_90Percentile | 0.0044 |  |
| AF_log-sigma-4-0-mm-3D_firstorder_Skewness | 0.0044 |  |
| AF_gradient_firstorder_InterquartileRange | 0.0044 |  |
| STAPES_wavelet-LLL_firstorder_Skewness | 0.0043 |  |
| AF_log-sigma-5-0-mm-3D_glrlm_GrayLevelNonUniformity | 0.0043 |  |
| AF_wavelet-HLL_firstorder_Mean | 0.0042 |  |
| AF_wavelet-LHH_gldm_DependenceNonUniformityNormalized | 0.0041 |  |
| AF_exponential_glrlm_GrayLevelNonUniformity | 0.0041 |  |
| AF_log-sigma-5-0-mm-3D_glszm_LargeAreaHighGrayLevelEmphasis | 0.0041 |  |
| AF_log-sigma-4-0-mm-3D_firstorder_10Percentile | 0.0040 |  |
| AF_square_glszm_GrayLevelNonUniformity | 0.0040 |  |

**Supplementary S6. Coefficients of the L2-Regularized Logistic Regression Model.** The best model was the LR trained on the 10 most significant features plus age, and sex. Presented below are the intercept and coefficient values of the regression model.

| Table S6. **Coefficients of the L2-Regularized Logistic Regression Model** | |
| --- | --- |
| **Feature** | **Coefficient** |
| Intercept | 0.06 |
| Age | -0.02 |
| Sex | 0.48 |
| AF_wavelet-LHH_firstorder_Mean | -0.58 |
| AF_log-sigma-1-0-mm-3D_firstorder_Mean | 0.11 |
| AF_gradient_firstorder_Median | -0.43 |
| AF_gradient_firstorder_Mean | -0.15 |
| AF_log-sigma-5-0-mm-3D_firstorder_Range | 0.52 |
| AF_original_firstorder_Mean | 0.08 |
| AF_logarithm_firstorder_10Percentile | -0.39 |
| AF_wavelet-LLL_firstorder_Mean | -0.32 |
| AF_original_firstorder_InterquartileRange | 0.08 |
| AF_gradient_firstorder_90Percentile | -0.11 |
| AF = Antefenestral region | |
